# Supplementary material for: Interrogation and validation of the interactome of neuronal Munc18-interacting Mint proteins with AlphaFold2
Source: J Biol Chem. 2023 Dec 9;300(1):105541. doi: 10.1016/j.jbc.2023.105541 (PMC10820826; doi:10.1016/j.jbc.2023.105541)
Supplement: Supplemental Tables [file mmc2.docx]

**Table S1. Putative interactors of human Mint1 from BioGRID^a^.**

| **Uniprot Entry ID** | **Gene Names** | **iPTM scores^b^** | **Average iPTM score^b^** | **AlphaFold predicted Interactor?^c^** | **Predicted interacting domains** | **References providing additional validation^d^** |
| --- | --- | --- | --- | --- | --- | --- |
| O14639 | ABLIM1 ABLIM KIAA0059 LIMAB1 | 0.24, 0.21, 0.21 | 0.22 | - |  |  |
| Q15067 | ACOX1 ACOX | 0.18, 0.18, 0.18 | 0.18 | - |  |  |
| Q9Y2T2 | AP3M1 | 0.54, 0.53, 0.51 | 0.53 | + | C-terminal β-sandwich platform domain of AP3 μ3 subunit and YxxΦ motif in Mint1 |  |
| P05067 | APP A4 AD1 | 0.32, 0.32, 0.31 | 0.32 | + | APP motif and Mint1 PTB domain. | (1-6) |
| P61204 | ARF3 | 0.83, 0.77, 0.18 | 0.59 | + | ARF3 small GTPase and Mint1 PDZ1-PDZ2 tandem domains. | (7) |
| P18085 | ARF4 | 0.83, 0.63, 0.55 | 0.67 | + | ARF4 small GTPase and Mint1 PDZ1-PDZ2 tandem domains. | (7) |
| Q96DR7 | ARHGEF26 SGEF HMFN1864 | 0.26, 0.25, 0.21 | 0.24 | - |  |  |
| Q9NXU5 | ARL15 ARFRP2 | 0.2, 0.19, 0.17 | 0.19 | - |  |  |
| Q9UHR4 | BAIAP2L1 IRTKS | 0.41, 0.17, 0.16 | 0.25 | - |  |  |
| Q14012 | CAMK1 | 0.22, 0.19, 0.16 | 0.19 | - |  |  |
| O14936 | CASK LIN2 | 0.46, 0.43, 0.43 | 0.44 | + | The CaM Kinase domain of CASK and the CID motif of Mint1 | (8-10) |
| O60563 | CCNT1 | 0.34, 0.22, 0.19 | 0.25 | - |  |  |
| O14618 | CCS | 0.41, 0.23, 0.22 | 0.29 | - |  | (11) |
| Q12834 | CDC20 | 0.22, 0.2, 0.19 | 0.20 | - |  |  |
| Q9P2B7 | CFAP97 KIAA1430 | 0.33, 0.19, 0.19 | 0.24 | - |  |  |
| Q9Y6M4 | CSNK1G3 | 0.37, 0.25, 0.24 | 0.29 | - |  |  |
| Q12959 | DLG1 | 0.22, 0.2, 0.19 | 0.20 | - |  | (12) |
| P78352 | DLG4 PSD95 | 0.22, 0.2, 0.17 | 0.20 | - |  |  |
| Q86SJ6 | DSG4 CDHF13 | 0.23, 0.22, 0.21 | 0.22 | - |  |  |
| Q9H8V3 | ECT2 | 0.25, 0.22, 0.21 | 0.23 | - |  |  |
| Q9H6Z9 | EGLN3 | 0.72, 0.6, 0.48 | 0.60 | - |  |  |
| Q8TE02 | ELP5 C17orf81 DERP6 HSPC002 MSTP071 | 0.19, 0.18, 0.16 | 0.18 | - |  |  |
| Q0PNE2 | ELP6 ATP1 C3orf75 TMEM103 | 0.64, 0.21, 0.2 | 0.35 | - |  |  |
| P49841 | GSK3B | 0.25, 0.24, 0.18 | 0.22 | - |  |  |
| Q9NYZ3 | GTSE1 | 0.23, 0.23, 0.22 | 0.23 | - |  |  |
| Q8WVV9 | HNRNPLL HNRPLL SRRF BLOCK24 | 0.19, 0.17, 0.17 | 0.18 | - |  |  |
| P17482 | HOXB9 HOX2E | 0.2, 0.2, 0.2 | 0.20 | - |  |  |
| P31274 | HOXC9 HOX3B | 0.21, 0.2, 0.19 | 0.20 | - |  |  |
| P28335 | HTR2C HTR1C | 0.27, 0.27, 0.26 | 0.27 | - |  | (13) |
| Q14500 | KCNJ12 IRK2 KCNJN1 | 0.25, 0.22, 0.22 | 0.23 | - | KCNJ12 motif and Mint1 PTB domain. | (12) |
| O60333 | KIF1B KIAA0591 KIAA1448 | 0.24, 0.24, 0.23 | 0.23 | - |  |  |
| O00139 | KIF2A KIF2 KNS2 | 0.18, 0.18, 0.16 | 0.17 | - |  |  |
| O95198 | KLHL2 | 0.19, 0.18, 0.18 | 0.18 | - |  |  |
| Q6TFL4 | KLHL24 DRE1 | 0.23, 0.21, 0.19 | 0.21 | - |  |  |
| Q9NS86 | LANCL2 GPR69B TASP | 0.42, 0.3, 0.18 | 0.3 | - |  |  |
| Q9UGP4 | LIMD1 | 0.21, 0.18, 0.17 | 0.19 | - |  |  |
| O14910 | LIN7A MALS1 VELI1 | 0.2, 0.19, 0.18 | 0.19 | - |  |  |
| Q9HAP6 | LIN7B MALS2 VELI2 UNQ3116/PRO10200 | 0.21, 0.19, 0.17 | 0.19 | - |  | (12) |
| Q9NUP9 | LIN7C MALS3 VELI3 | 0.22, 0.23, 0.17 | 0.20 | - |  | (12) |
| Q14114 | LRP8 APOER2 | 0.31, 0.3, 0.3 | 0.30 | + | LRP8 motif and Mint2 PTB domain. | (14) |
| Q9P0L2 | MARK1 KIAA1477 MARK | 0.28, 0.25, 0.2 | 0.24 | - |  |  |
| Q7L590 | MCM10 PRO2249 | 0.19, 0.19, 0.18 | 0.19 | - |  |  |
| Q9BTE3 | MCMBP C10orf119 | 0.47, 0.38, 0.27 | 0.37 | - |  |  |
| Q8WV92 | MITD1 | 0.21, 0.2, 0.15 | 0.19 | - |  |  |
| P61764 | Munc18-1 STXBP1 UNC18A | 0.68, 0.67, 0.6 | 0.65 | + | Munc18-1 domain3a and Mint1 AHM. | (15-22). This study. |
| Q7Z628 | NET1 ARHGEF8 | 0.42, 0.37, 0.25 | 0.35 | - |  |  |
| Q12986 | NFX1 NFX2 | 0.33, 0.25, 0.21 | 0.26 | - |  |  |
| P52952 | NKX2-5 CSX NKX2.5 NKX2E | 0.21, 0.19, 0.17 | 0.19 | - |  |  |
| Q9BW91 | NUDT9 NUDT10 PSEC0099 UNQ3012/PRO9771 | 0.31, 0.19, 0.16 | 0.22 | - |  |  |
| O95747 | OXSR1 KIAA1101 OSR1 | 0.32, 0.26, 0.25 | 0.28 | - |  |  |
| Q6ZW49 | PAXIP1 PAXIP1L PTIP CAGF28 | 0.36, 0.27, 0.26 | 0.30 | - |  |  |
| Q13526 | PIN1 | 0.19, 0.19, 0.17 | 0.18 | - |  |  |
| Q99755 | PIP5K1A | 0.19, 0.18, 0.16 | 0.18 | - |  |  |
| Q99569 | PKP4 | 0.39, 0.37, 0.31 | 0.36 | - |  |  |
| O00411 | POLRMT | 0.27, 0.26, 0.24 | 0.26 | - |  |  |
| Q13356 | PPIL2 | 0.26, 0.21, 0.21 | 0.23 | - |  |  |
| Q9BZL6 | PRKD2 PKD2 HSPC187 | 0.25, 0.2, 0.2 | 0.22 | - |  |  |
| P49810 | PSEN2 AD4 PS2 PSNL2 STM2 | 0.22, 0.18, 0.17 | 0.19 | - |  | (1) |
| Q5TGL8 | PXDC1 C6orf145 | 0.37, 0.2, 0.19 | 0.25 | - |  |  |
| Q9NW64 | RBM22 ZC3H16 199G4 | 0.24, 0.23, 0.2 | 0.22 | - |  |  |
| O94761 | RECQL4 RECQ4 | 0.32, 0.27, 0.23 | 0.27 | - |  |  |
| Q8WVD3 | RNF138 NARF HSD-4 HSD4 | 0.19, 0.19, 0.17 | 0.18 | - |  |  |
| Q96P16 | RPRD1A P15RS | 0.27, 0.25, 0.22 | 0.25 | - |  |  |
| P51812 | RPS6KA3 ISPK1 MAPKAPK1B RSK2 | 0.23, 0.23, 0.23 | 0.23 | - |  |  |
| Q7L4I2 | RSRC2 | 0.22, 0.18, 0.17 | 0.19 | - |  |  |
| P33764 | S100A3 S100E | 0.33, 0.32, 0.2 | 0.28 | - |  |  |
| Q86UD0 | SAPCD2 C9orf140 | 0.29, 0.27, 0.27 | 0.28 | - |  |  |
| Q9BXP2 | SLC12A9 CCC6 CIP1 | 0.21, 0.19, 0.17 | 0.19 | - |  |  |
| Q8N5C6 | SRBD1 | 0.3, 0.21, 0.19 | 0.23 | - |  |  |
| Q8WVM0 | TFB1M CGI-75 | 0.2, 0.19, 0.15 | 0.18 | - |  |  |
| Q5JTD0 | TJAP1 PILT TJP4 | 0.19, 0.19, 0.16 | 0.18 | - |  |  |
| O95985 | TOP3B TOP3B1 | 0.32, 0.22, 0.23 | 0.26 | - |  |  |
| Q9ULW0 | TPX2 C20orf1 C20orf2 DIL2 HCA519 | 0.27, 0.25, 0.17 | 0.23 | - |  |  |
| Q8WVR3 | TRAPPC14 C7orf43 MAP11 | 0.26, 0.26, 0.18 | 0.23 | - |  |  |
| Q9Y296 | TRAPPC4 SBDN CGI-104 HSPC172 PTD009 | 0.38, 0.21, 0.16 | 0.25 | - |  |  |
| Q96Q05 | TRAPPC9 KIAA1882 NIBP T1 | 0.23, 0.22, 0.19 | 0.21 | - |  |  |
| Q7Z2T5 | TRMT1L C1orf25 TRM1L MSTP070 | 0.19, 0.18, 0.17 | 0.18 | - |  |  |
| Q9UPT9 | USP22 KIAA1063 USP3L | 0.39, 0.28, 0.27 | 0.31 | - |  |  |
| Q8IWA0 | WDR75 UTP17 | 0.25, 0.19, 0.19 | 0.21 | - |  |  |
| O95785 | WIZ ZNF803 | 0.62, 0.62, 0.36 | 0.53 | + | C-terminal Zinc-finger of WIZ and the PDZ1-PDZ2 tandem domains of Mint1 |  |
| Q86U90 | YRDC DRIP3 IRIP | 0.32, 0.23, 0.22 | 0.26 | - |  |  |
| Q8N5A5 | ZGPAT GPATC6 GPATCH6 KIAA1847 ZC3H9 ZC3HDC9 ZIP | 0.23, 0.21, 0.17 | 0.20 | - |  |  |

a. <https://thebiogrid.org/106817/summary/homo-sapiens/apba1.html>

b. Individual iPTM scores and average iPTM scores are provided for the three predicted models.

c. The definition of “Interactor” is based on a semi-quantitative measure of the AlphaFold2-derived interfacial PTM (iPTM) score, visual inspection of the resultant PAE graphs and assessment of the aligned structures in PyMOL. After generating three separate multimer predictions in AlphaFold2 in unsupervised batch mode, we found that a minimum average iPTM score of 0.3 combined with a strong signal in the PAE plot for inter-molecular structural correlation provided a useful indicator for structural predictions that merited further assessment. In these cases, we then ran three independent modelling experiments focused on the interacting regions to assess whether multiple predictions resulted in consistent structural alignments in PyMOL.

d. The BioGRID database provides references for each identified putative interaction. These are typically, although not always, high-throughput proteomics studies. Here we provide citations for other studies that have provided additional validation using methods such as immunoprecipitation, *in vitro* binding assays, or macromolecular structure determination.

**Table S2. Putative interactors of human Mint2 from BioGRID^a^.**

| **Uniprot Entry ID** | **Gene Names** | **iPTM scores^b^** | **Average iPTM score^b^** | **AlphaFold predicted Interactor?^c^** | **Predicted interacting domains** | **References providing additional validation^d^** |
| --- | --- | --- | --- | --- | --- | --- |
| Q6PD74 | AAGAB | 0.17, 0.16, 0.16 | 0.16 | - |  |  |
| Q9UBZ4 | APEX2 APE2 APEXL2 XTH2 | 0.23, 0.22, 0.18 | 0.21 | - |  |  |
| P05067 | APP A4 AD1 | 0.84, 0.83, 0.80 | 0.82 | + | APP motif and Mint2 PTB domain. | (2, 3, 5, 6) |
| P61204 | ARF3 | 0.84, 0.81, 0.81 | 0.82 | + | ARF3 small GTPase and Mint2 PDZ1-PDZ2 tandem domains. | (7) |
| P18085 | ARF4 | 0.87, 0.85, 0.84 | 0.85 | + | ARF4 small GTPase and Mint2 PDZ1-PDZ2 tandem domains. | (7) |
| Q7Z3C6 | ATG9A APG9L1 | 0.32, 0.3, 0.28 | 0.30 | - |  |  |
| O15155 | BET1 | 0.22, 0.21, 0.15 | 0.19 | - |  |  |
| Q9Y297 | BTRC BTRCP FBW1A FBXW1A | 0.37, 0.32, 0.15 | 0.28 | - |  |  |
| A6NLJ0 | C2CD4B FAM148B NLF2 | 0.27, 0.2, 0.18 | 0.22 | - |  |  |
| O94985 | CLSTN1 CS1 KIAA0911 | 0.34, 0.34, 0.33 | 0.34 | + | CLSTN1 motif and Mint2 PTB domain. | (23) |
| Q9GZR7 | DDX24 | 0.19, 0.19, 0.19 | 0.19 | - |  |  |
| Q09019 | DMWD DM9 | 0.41, 0.4, 0.29 | 0.37 | - |  |  |
| Q9UKB1 | FBXW11 BTRCP2 FBW1B FBXW1B KIAA0696 | 0.33, 0.19, 0.16 | 0.23 | - |  |  |
| A4D1S0 | KLRG2 CLEC15B | 0.19, 0.18, 0.16 | 0.18 | - |  |  |
| Q07954 | LRP1 A2MR APR | 0.82, 0.82, 0.78 | 0.81 | + | LRP1 motif and Mint2 PTB domain. | (24) |
| P98164 | LRP2 | 0.61, 0.59, 0.55 | 0.59 | + | LRP2 motif and Mint2 PTB domain. | (24) |
| Q14114 | LRP8 APOER2 | 0.81, 0.77, 0.34 | 0.64 | + | LRP8 motif and Mint2 PTB domain. | (24) |
| Q8N7X4 | MAGEB6 | 0.38, 0.34, 0.33 | 0.35 | - |  |  |
| Q16659 | MAPK6 ERK3 PRKM6 | 0.26, 0.26, 0.19 | 0.24 | - |  |  |
| P61764 | Munc18-1 STXBP1 UNC18A | 0.67, 0.66, 0.63 | 0.65 | + | Munc18-1 domain3a and Mint2 AHM. | (15-22). This study. |
| Q96P71 | NECAB3 APBA2BP NIP1 SYTIP2 XB51 | 0.16, 0.16, 0.14 | 0.15 | - |  | (25) |
| Q13393 | PLD1 | 0.27, 0.22, 0.2 | 0.23 | - |  |  |
| P49810 | PSEN2 AD4 PS2 PSNL2 STM2 | 0.16, 0.15, 0.15 | 0.15 | - |  | (1) |
| O94761 | RECQL4 RECQ4 | 0.28, 0.28, 0.26 | 0.27 | - |  |  |
| Q04206 | RELA NFKB3 | 0.23, 0.18, 0.17 | 0.19 | - |  | (26) |
| Q9NVW2 | RLIM RNF12 | 0.22, 0.2, 0.19 | 0.20 | - |  |  |
| Q68DV7 | RNF43 | 0.32, 0.28, 0.25 | 0.28 | - |  |  |
| O15431 | SLC31A1 COPT1 CTR1 | 0.27, 0.2, 0.18 | 0.22 | - |  |  |
| Q9HB58 | SP110 | 0.17, 0.17, 0.17 | 0.17 | - |  |  |
| Q8N9I0 | SYT2 | 0.17, 0.15, 0.15 | 0.16 | - |  |  |
| Q5JTD0 | TJAP1 PILT TJP4 | 0.40, 0.31, 0.29 | 0.33 | + | TJAP1 N-terminal sequence and Mint2 PTB domain in non-canonical site |  |
| Q8NFZ5 | TNIP2 ABIN2 FLIP1 | 0.16, 0.15, 0.14 | 0.15 | - |  |  |
| Q8WV44 | TRIM41 RINCK | 0.19, 0.17, 0.17 | 0.18 | - |  |  |
| Q9Y2K6 | USP20 KIAA1003 LSFR3A VDU2 | 0.32, 0.28, 0.24 | 0.28 | - |  |  |
| Q8TEY7 | USP33 KIAA1097 VDU1 | 0.29, 0.27, 0.25 | 0.27 | - |  |  |
| Q6AHZ1 | ZNF518A KIAA0335 ZNF518 | 0.23, 0.23, 0.16 | 0.21 | - |  |  |
| Q6DN90 | IQSEC1 ARFGEP100  BRAG2 KIAA0763 | 0.79, 0.78, 0.77 | 0.77 | + | IQSEC1 PH domain and peptide sequence in Mint2 N-terminal region |  |
| Q5JU85 | IQSEC2  KIAA0522 | 0.66, 0.65, 0.59 | 0.65 | + | IQSEC2  PH domain and peptide sequence in Mint2 N-terminal region |  |
| Q5VYV7 | SLX4IP  C20orf94 | 0.19, 0.19, 0.16 | 0.18 | - |  |  |

a. <https://thebiogrid.org/106817/summary/homo-sapiens/apba1.html>

b. Individual iPTM scores and average iPTM scores are provided for the three predicted models.

c. The definition of “Interactor” is based on a semi-quantitative measure of the AlphaFold2-derived interfacial PTM (iPTM) score, visual inspection of the resultant PAE graphs and assessment of the aligned structures in PyMOL. After generating three separate multimer predictions in AlphaFold2 in unsupervised batch mode, we found that a minimum average iPTM score of 0.3 combined with a strong signal in the PAE plot for inter-molecular structural correlation provided a useful indicator for structural predictions that merited further assessment. In these cases, we then ran three independent modelling experiments focused on the interacting regions to assess whether multiple predictions resulted in consistent structural alignments in PyMOL.

d. The BioGRID database provides references for each identified putative interaction. These are typically, although not always, high-throughput proteomics studies. Here we provide citations for other studies that have provided additional validation using methods such as immunoprecipitation, *in vitro* binding assays, or macromolecular structure determination.

**Table S3. Resources and reagents.**

| **REAGENT or RESOURCE** | **SOURCE or REFERENCE** | **IDENTIFIER** |
| --- | --- | --- |
| **Bacterial Strains** | | |
| *E. coli* DH5α | Invitrogen | 18265017 |
| *E. coli* Rosetta™(DE3) | Merck Australia | 70954 |
|  |  |  |
| **Cell Lines** | | |
| Pheochromocytoma cells (PC12) | ATCC | CRL-1721 |
|  |  |  |
| **Antibodies** | | |
| GFP polyclonal antibody | Thermo Fisher | A6455 |
| BD Mouse Monoclonal anti-Munc18-1 | BD Biosciences | 610336 |
| Goat anti mouse secondary antibody | Thermo Fisher | 65-6120 |
|  |  |  |
| **Chemicals** | | |
| Benzamidine hydrochloride hydrate | Sigma Aldrich | B6506 |
| Deoxyribonuclease I (DNase I) | Sigma Aldrich | DN25 |
| Talon® resin | Clontech | 635503 |
| Glutathione Sepharose 4B | GE Healthcare | GEHE17-0756-0 |
| Isopropyl β-D-1-thiogalactopyranoside | Bioline | BIO-37036 |
| LipofectamineTM LTX with PLUS ReagentTM | ThermoFisher | 15338-100 |
| Poly-D-Lysine | SigmaAldrich | P2636-100MG |
| Glass-bottom petri-dishes | Cellvis | D29-20-1.5-N |
|  |  |  |
| **Recombinant DNA** | | |
| Plasmid: pGEX4T-2 | Cytiva | 28-9545-50 |
| Plasmid: pGEX4T-2 GST- Mint1(226-314)(MID) | This study | N/A |
| Plasmid: pGEX4T-2 GST- Mint1(261-272) | This study | N/A |
| Plasmid: pGEX4T-2 GST- Mint1(261-282) | This study | N/A |
| Plasmid: pGEX4T-2 GST- Mint1(226-289) | This study | N/A |
| Plasmid: pGEX4T-2 GST- Mint1(222-314) | This study | N/A |
| Plasmid: pGEX4T-2 GST- Mint1(237-289) | This study | N/A |
| Plasmid: pcDNA3.1-N-eGFP Mint1-GFP | This study | N/A |
| Plasmid: pcDNA3.1-N-eGFP Mint1(D269A/I270A)-GFP | This study | N/A |
| Plasmid: pET24a(+) Sx1a_1-261_-His | (27) | N/A |
| Plasmid: pET28a(+) Munc18-1-His | (28) | N/A |
| Plasmid: pET28a(+) Munc18-1^Δ317-333^-His | (29) | N/A |
| pEGFP-N1 | Clontec | 6085-1 |
| pmEos3.2-N1 Munc18-1^WT^ | This study | N/A |
| pmEos3.2-N1 Munc18-1^R388A^ | This study | N/A |
| pCI VAMP2-pHluorin | (30) | N/A |
|  |  |  |
| **Deposited Data** | | |
| Minc18-1/Mint1/Sx1 complex (crystal structure) | RSCB Protein Data Bank | PDB ID: 7XSJ |
| Human Mint1 (protein sequence) | Uniprot | Q02410-1 |
| Human Mint1 (open reading frame) | Genscript | NM_001163.3 |
| Rat Munc18-1 (protein sequence) | Uniprot | P61765-1 |
| Human Sx1a (protein sequence | Uniprot | Q16623-1 |
| Human Mint1 interactome | BioGrid | https://thebiogrid.org/106817/summary/homo-sapiens/apba1.html |
| Human Mint2 interactome | BioGrid | https://thebiogrid.org/106818/summary/homo-sapiens/apba2.html |
|  |  |  |
| **Software** | | |
| Pymol | Schrodinger, USA. | https://pymol.org/2/ |
| BioGrid4.4.217 | (31) | https://thebiogrid.org |
| Consurf | (32) | https://consurf.tau.ac.il/consurf_index.php |
| AlphaFold2 Multimer | (33, 34) | https://github.com/deepmind/alphafold |
| ColabFold and ColabFold batch | (35) | https://github.com/sokrypton/ColabFold |
| Fiji/ImageJ (version 2.9.0) | (36) | https://imagej.net/software/fiji/downloads |
| Python 3.8 | N/A | https://www.python.org/downloads/release/python-380/ |
| DBSCAN | (37) | https://scikit-learn.org/stable/modules/generated/sklearn.cluster.DBSCAN.html |
|  |  |  |
| **Other** | | |
| Superose6 Increase10/300 GL | GE Healthcare | Catalogue: 29091596 |
| HiLoad^TM^ Superdex75 PG | GE Healthcare | Catalogue: 28989333 |

**References**

1. Biederer, T., Cao, X., Sudhof, T. C., andLiu, X. (2002) Regulation of APP-dependent transcription complexes by Mint/X11s: differential functions of Mint isoforms J Neurosci **22**, 7340-7351, <https://www.ncbi.nlm.nih.gov/pubmed/12196555>

2. Borg, J. P., Ooi, J., Levy, E., andMargolis, B. (1996) The phosphotyrosine interaction domains of X11 and FE65 bind to distinct sites on the YENPTY motif of amyloid precursor protein Mol Cell Biol **16**, 6229-6241 10.1128/mcb.16.11.6229

3. Matos, M. F., Xu, Y., Dulubova, I., Otwinowski, Z., Richardson, J. M., Tomchick, D. R. *et al.* (2012) Autoinhibition of Mint1 adaptor protein regulates amyloid precursor protein binding and processing Proc Natl Acad Sci U S A **109**, 3802-3807 10.1073/pnas.1119075109

4. Sakuma, M., Tanaka, E., Taru, H., Tomita, S., Gandy, S., Nairn, A. C. *et al.* (2009) Phosphorylation of the amino-terminal region of X11L regulates its interaction with APP J Neurochem **109**, 465-475 10.1111/j.1471-4159.2009.05988.x

5. Xie, X., Yan, X., Wang, Z., Zhou, H., Diao, W., Zhou, W. *et al.* (2013) Open-closed motion of Mint2 regulates APP metabolism J Mol Cell Biol **5**, 48-56 10.1093/jmcb/mjs033

6. Zhang, Z., Lee, C. H., Mandiyan, V., Borg, J. P., Margolis, B., Schlessinger, J. *et al.* (1997) Sequence-specific recognition of the internalization motif of the Alzheimer's amyloid precursor protein by the X11 PTB domain EMBO J **16**, 6141-6150 10.1093/emboj/16.20.6141

7. Hill, K., Li, Y., Bennett, M., McKay, M., Zhu, X., Shern, J. *et al.* (2003) Munc18 interacting proteins: ADP-ribosylation factor-dependent coat proteins that regulate the traffic of beta-Alzheimer's precursor protein J Biol Chem **278**, 36032-36040 10.1074/jbc.M301632200

8. Wu, X., Cai, Q., Chen, Y., Zhu, S., Mi, J., Wang, J. *et al.* (2020) Structural Basis for the High-Affinity Interaction between CASK and Mint1 Structure **28**, 664-673 e663 10.1016/j.str.2020.04.001

9. Zhang, Z., Li, W., Yang, G., Lu, X., Qi, X., Wang, S. *et al.* (2020) CASK modulates the assembly and function of the Mint1/Munc18-1 complex to regulate insulin secretion Cell Discov **6**, 92 10.1038/s41421-020-00216-3

10. Borg, J. P., Straight, S. W., Kaech, S. M., de Taddeo-Borg, M., Kroon, D. E., Karnak, D. *et al.* (1998) Identification of an evolutionarily conserved heterotrimeric protein complex involved in protein targeting J Biol Chem **273**, 31633-31636 10.1074/jbc.273.48.31633

11. McLoughlin, D. M., Standen, C. L., Lau, K. F., Ackerley, S., Bartnikas, T. P., Gitlin, J. D. *et al.* (2001) The neuronal adaptor protein X11alpha interacts with the copper chaperone for SOD1 and regulates SOD1 activity J Biol Chem **276**, 9303-9307 10.1074/jbc.M010023200

12. Leonoudakis, D., Conti, L. R., Radeke, C. M., McGuire, L. M., andVandenberg, C. A. (2004) A multiprotein trafficking complex composed of SAP97, CASK, Veli, and Mint1 is associated with inward rectifier Kir2 potassium channels J Biol Chem **279**, 19051-19063 10.1074/jbc.M400284200

13. Becamel, C., Alonso, G., Galeotti, N., Demey, E., Jouin, P., Ullmer, C. *et al.* (2002) Synaptic multiprotein complexes associated with 5-HT(2C) receptors: a proteomic approach EMBO J **21**, 2332-2342 10.1093/emboj/21.10.2332

14. He, X., Cooley, K., Chung, C. H., Dashti, N., andTang, J. (2007) Apolipoprotein receptor 2 and X11 alpha/beta mediate apolipoprotein E-induced endocytosis of amyloid-beta precursor protein and beta-secretase, leading to amyloid-beta production J Neurosci **27**, 4052-4060 10.1523/JNEUROSCI.3993-06.2007

15. Biederer, T., andSudhof, T. C. (2000) Mints as adaptors. Direct binding to neurexins and recruitment of munc18 J Biol Chem **275**, 39803-39806 10.1074/jbc.C000656200

16. Graham, M. E., Prescott, G. R., Johnson, J. R., Jones, M., Walmesley, A., Haynes, L. P. *et al.* (2011) Structure-function study of mammalian Munc18-1 and C. elegans UNC-18 implicates domain 3b in the regulation of exocytosis PLoS One **6**, e17999 10.1371/journal.pone.0017999

17. Han, G. A., Park, S., Bin, N. R., Jung, C. H., Kim, B., Chandrasegaram, P. *et al.* (2014) A pivotal role for pro-335 in balancing the dual functions of Munc18-1 domain-3a in regulated exocytosis J Biol Chem **289**, 33617-33628 10.1074/jbc.M114.584805

18. Ho, C. S., Marinescu, V., Steinhilb, M. L., Gaut, J. R., Turner, R. S., andStuenkel, E. L. (2002) Synergistic effects of Munc18a and X11 proteins on amyloid precursor protein metabolism J Biol Chem **277**, 27021-27028 10.1074/jbc.M201823200

19. Okamoto, M., andSudhof, T. C. (1997) Mints, Munc18-interacting proteins in synaptic vesicle exocytosis J Biol Chem **272**, 31459-31464 10.1074/jbc.272.50.31459

20. Okamoto, M., andSudhof, T. C. (1998) Mint 3: a ubiquitous mint isoform that does not bind to munc18-1 or -2 Eur J Cell Biol **77**, 161-165 10.1016/S0171-9335(98)80103-9

21. Park, J. H., Jung, M. S., Kim, Y. S., Song, W. J., andChung, S. H. (2012) Phosphorylation of Munc18-1 by Dyrk1A regulates its interaction with Syntaxin 1 and X11alpha J Neurochem **122**, 1081-1091 10.1111/j.1471-4159.2012.07861.x

22. Li, W., Xing, Y., Wang, Y., Xu, T., Song, E., andFeng, W. (2023) A non-canonical target-binding site in Munc18-1 domain 3b for assembling the Mint1-Munc18-1-syntaxin-1 complex Structure **31**, 68-77 e65 10.1016/j.str.2022.11.002

23. Araki, Y., Tomita, S., Yamaguchi, H., Miyagi, N., Sumioka, A., Kirino, Y. *et al.* (2003) Novel cadherin-related membrane proteins, Alcadeins, enhance the X11-like protein-mediated stabilization of amyloid beta-protein precursor metabolism J Biol Chem **278**, 49448-49458 10.1074/jbc.M306024200

24. Gotthardt, M., Trommsdorff, M., Nevitt, M. F., Shelton, J., Richardson, J. A., Stockinger, W. *et al.* (2000) Interactions of the low density lipoprotein receptor gene family with cytosolic adaptor and scaffold proteins suggest diverse biological functions in cellular communication and signal transduction J Biol Chem **275**, 25616-25624 10.1074/jbc.M000955200

25. Lee, D. S., Tomita, S., Kirino, Y., andSuzuki, T. (2000) Regulation of X11L-dependent amyloid precursor protein metabolism by XB51, a novel X11L-binding protein J Biol Chem **275**, 23134-23138 10.1074/jbc.C000302200

26. Tomita, S., Fujita, T., Kirino, Y., andSuzuki, T. (2000) PDZ domain-dependent suppression of NF-kappaB/p65-induced Abeta42 production by a neuron-specific X11-like protein J Biol Chem **275**, 13056-13060 10.1074/jbc.c000019200

27. Christie, M. P., Whitten, A. E., King, G. J., Hu, S. H., Jarrott, R. J., Chen, K. E. *et al.* (2012) Low-resolution solution structures of Munc18:Syntaxin protein complexes indicate an open binding mode driven by the Syntaxin N-peptide Proc Natl Acad Sci U S A **109**, 9816-9821 10.1073/pnas.1116975109

28. Hu, S. H., Christie, M. P., Saez, N. J., Latham, C. F., Jarrott, R., Lua, L. H. *et al.* (2011) Possible roles for Munc18-1 domain 3a and Syntaxin1 N-peptide and C-terminal anchor in SNARE complex formation Proc Natl Acad Sci U S A **108**, 1040-1045 10.1073/pnas.0914906108

29. Martin, S., Tomatis, V. M., Papadopulos, A., Christie, M. P., Malintan, N. T., Gormal, R. S. *et al.* (2013) The Munc18-1 domain 3a loop is essential for neuroexocytosis but not for syntaxin-1A transport to the plasma membrane J Cell Sci **126**, 2353-2360 10.1242/jcs.126813

30. Miesenbock, G., De Angelis, D. A., andRothman, J. E. (1998) Visualizing secretion and synaptic transmission with pH-sensitive green fluorescent proteins Nature **394**, 192-195 10.1038/28190

31. Oughtred, R., Rust, J., Chang, C., Breitkreutz, B. J., Stark, C., Willems, A. *et al.* (2021) The BioGRID database: A comprehensive biomedical resource of curated protein, genetic, and chemical interactions Protein Sci **30**, 187-200 10.1002/pro.3978

32. Ashkenazy, H., Abadi, S., Martz, E., Chay, O., Mayrose, I., Pupko, T. *et al.* (2016) ConSurf 2016: an improved methodology to estimate and visualize evolutionary conservation in macromolecules Nucleic Acids Res **44**, W344-350 10.1093/nar/gkw408

33. Jumper, J., Evans, R., Pritzel, A., Green, T., Figurnov, M., Ronneberger, O. *et al.* (2021) Highly accurate protein structure prediction with AlphaFold Nature **596**, 583-589 10.1038/s41586-021-03819-2

34. Evans, R., O’Neill, M., Pritzel, A., Antropova, N., Senior, A., Green, T. *et al.* (2022) Protein complex prediction with AlphaFold-Multimer bioRxiv 2021.2010.2004.463034 10.1101/2021.10.04.463034

35. Mirdita, M., Schutze, K., Moriwaki, Y., Heo, L., Ovchinnikov, S., andSteinegger, M. (2022) ColabFold: making protein folding accessible to all Nat Methods **19**, 679-682 10.1038/s41592-022-01488-1

36. Schneider, C. A., Rasband, W. S., andEliceiri, K. W. (2012) NIH Image to ImageJ: 25 years of image analysis Nat Methods **9**, 671-675 10.1038/nmeth.2089

37. Schubert, E., Sander, J., Ester, M., Kriegel, H. P., andXu, X. (2017) DBSCAN Revisited, Revisited: Why and How You Should (Still) Use DBSCAN ACM Trans Database Syst **42**, Article 19 10.1145/3068335
