# Supplementary material for: Interrogation and validation of the interactome of neuronal Munc18-interacting Mint proteins with AlphaFold2
Source: J Biol Chem. 2023 Dec 9;300(1):105541. doi: 10.1016/j.jbc.2023.105541 (PMC10820826; doi:10.1016/j.jbc.2023.105541)
Supplement: Supplemental Figures [file mmc3.pdf]

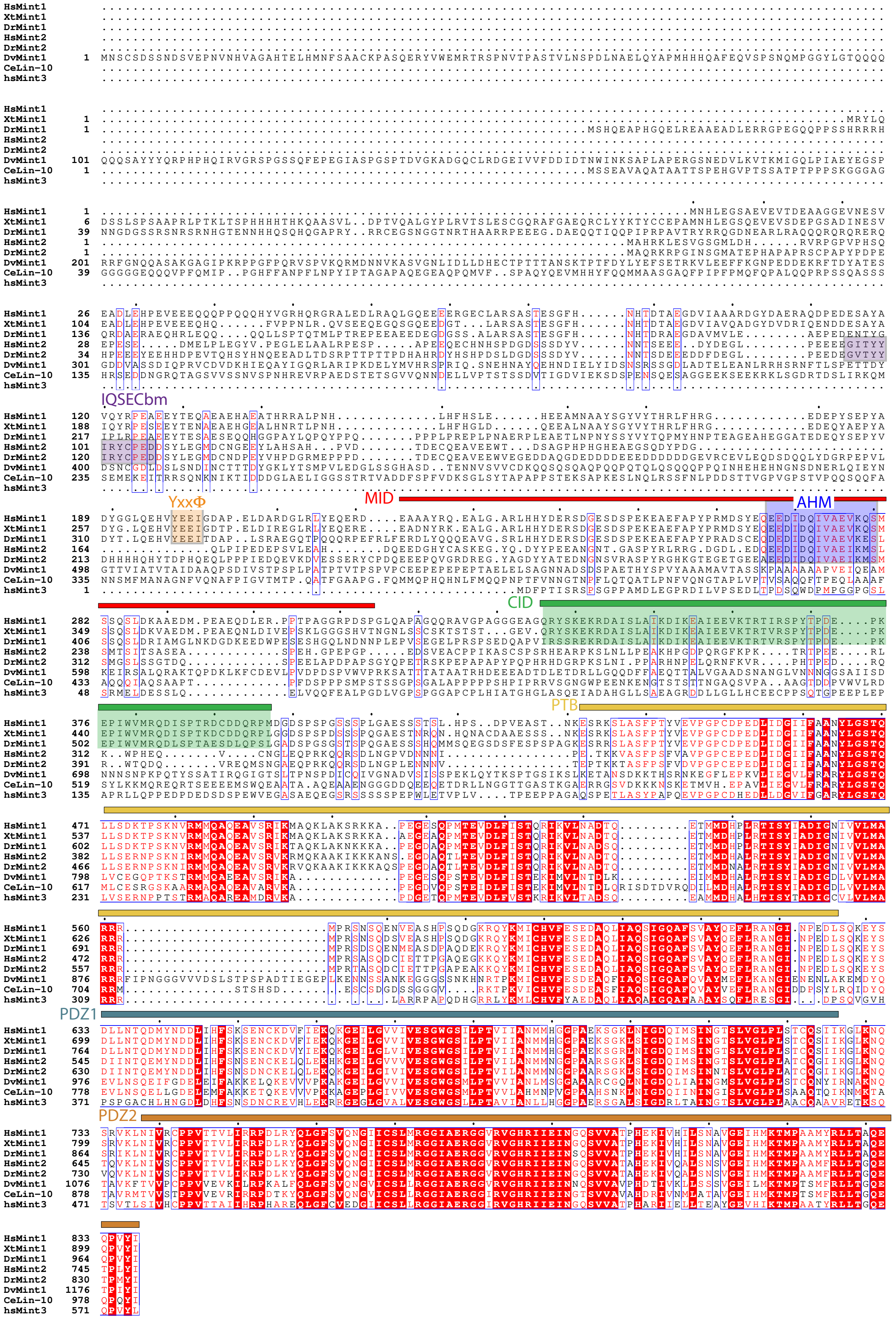

Figure S1. Sequence alignment of Mint proteins. (Related to Fig. 1)

(A) Sequence alignment Mint1, Mint2 and Mint3. Alignment was performed with Multalin (Corpet et al, 1999) and rendered with ESPript3.0 (Robert & Gouet, 2014). Hs, Homo sapiens. Xt, Xenopus tropicalis. Dr, Danio rerio. Ce, Caenorhabditis elegans. Dv, Drosophila virilis.

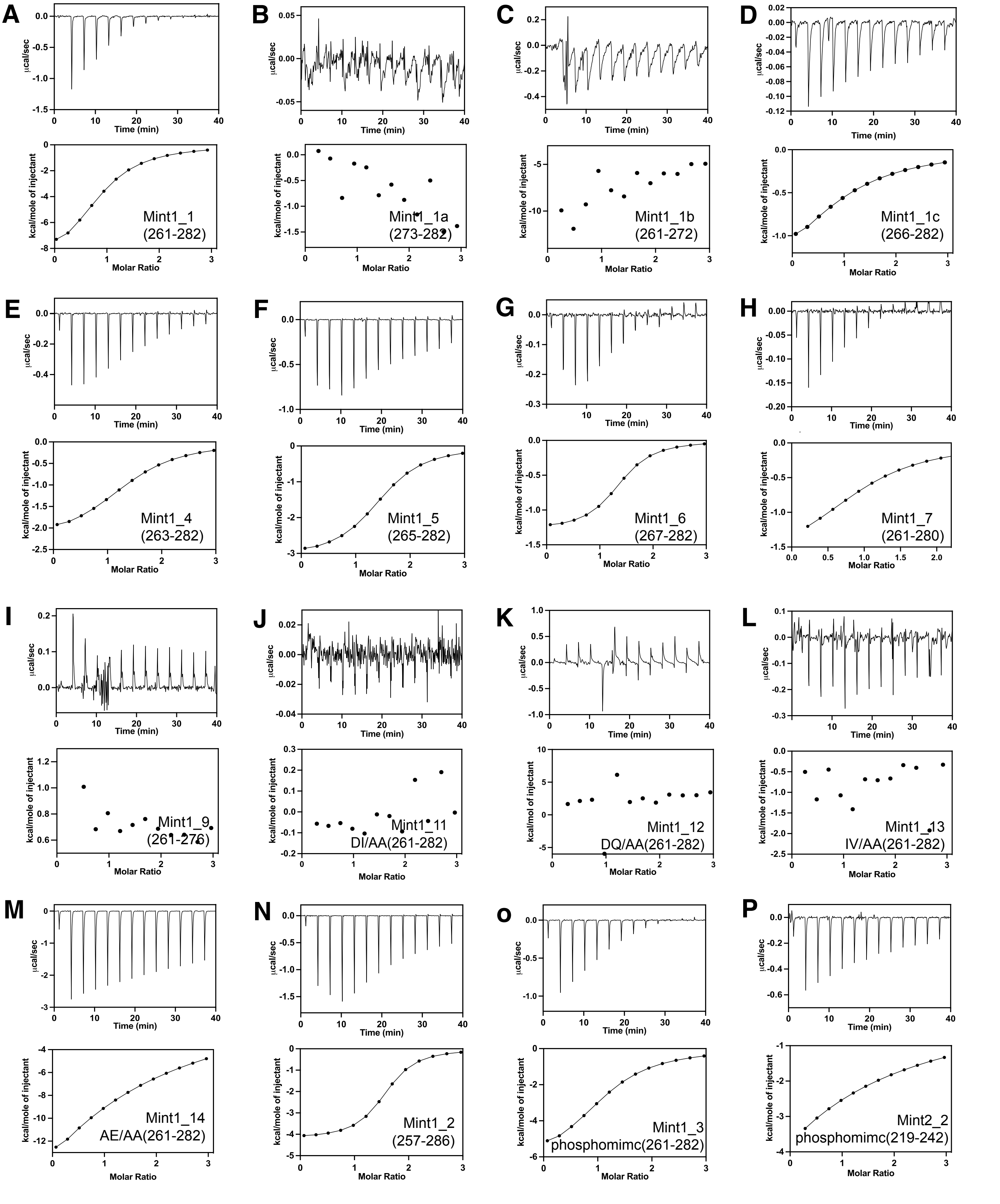

Figure S2. ITC experiments of Munc18-1 binding to various Mint peptides. (Related to Fig. 2)

Example ITC experiments are shown for each of the Mint peptides binding to Munc18-1 as described in Fig. 2B and Table 1. (A) Mint 1_1 (261-282) (B) Mint 1_1a (273-282) (C) Mint 1_1b (261-272) (D) Mint 1_1c (266-282) (E) Mint 1_4 (263-282) (F) Mint 1_5 (265-282) (G) Mint 1_6 (267-282) (H) Mint 1_7 (261-280) (I) Mint 1_9 (261-276) (J) Mint 1_11 DI /AA (261-282 (K) Mint 1-12 DQ /AA (261-282) (L) Mint 1_13 IV/AA(261-282) (M) Mint 1_14 AE/AA(261-282) (N) Mint 1_2 (257-286) (O) Mint 1_3 phosphomimetic (261-282) (P) Mint 2_2 phosphomimetic (219-242). Table 1 shows the binding affinities (Kd) of the above Mint 1 peptides obtained by integrating and normalising data fit with 1:1 ratio binding model. The Kd for Mint 1-1, 1a,1b and 1c are given as a mean of at least 2 independent experiments (n=2) and for all the other peptides Kds have been calculated from a single experiment (n=1). The peptides for which binding was not detected are shown as 'nb,' abbreviating 'No Binding was detected".

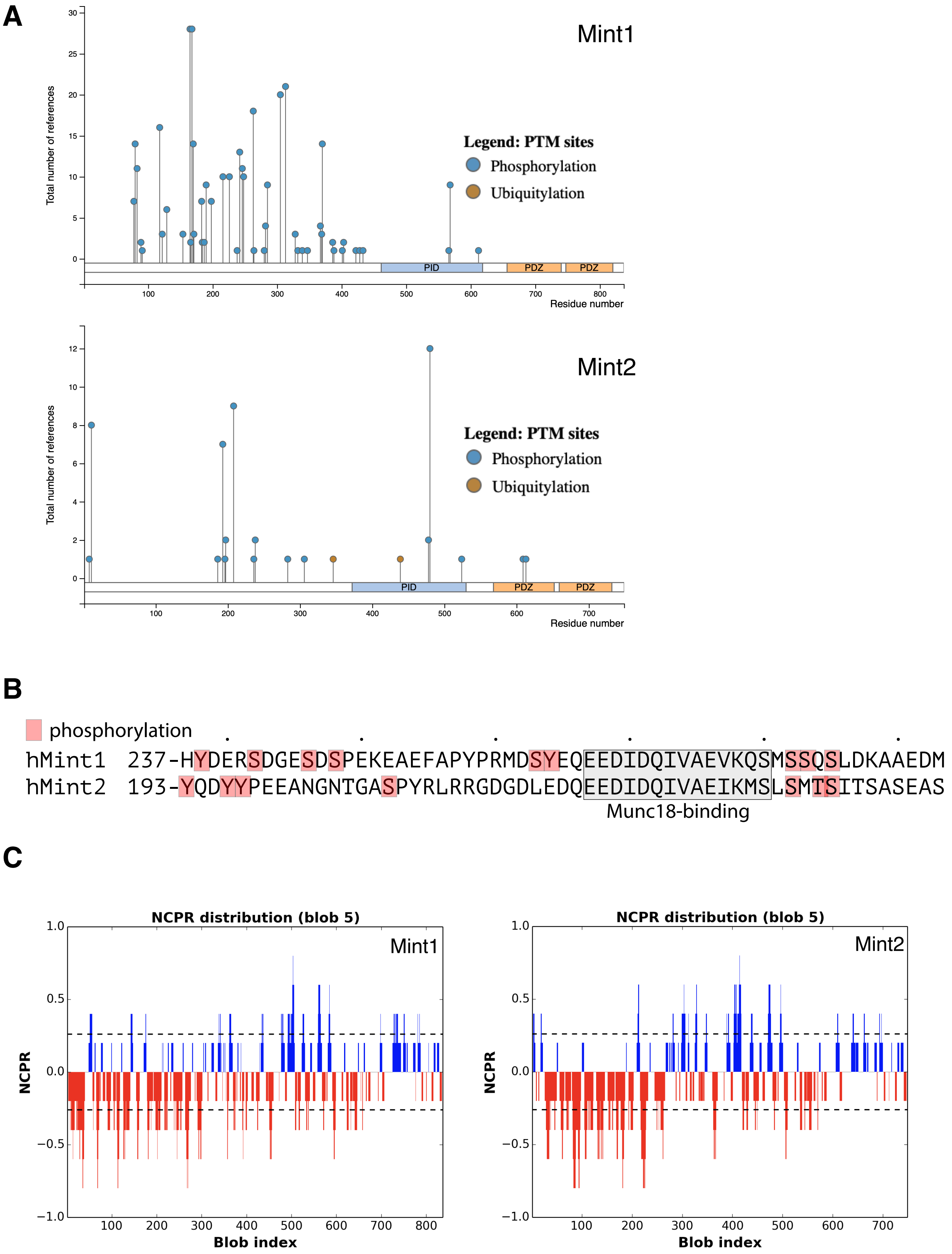

Figure S3. Post-translational modifications and sequence charge distribution of human Mint1 and Mint2. (Related to Fig. 2)
(A) Sites of phosphorylation and ubiquitination of human Mint1 and Mint2 experimentally observed and reported in PhosphoSitePlus (Hornbeck et al., 2015). (B) Phosphorylation sites in the human Mint1 and Mint2 N-terminal regions documented in PhosphoSitePlus (Hornbeck et al., 2015). Phosphomimetic mutations in regions adjacent to the Munc18-1 binding sequence do not influence affinity (Fig. 2B; Table 1). (C) Plot of the charge distribution versus the sequence of Mint1 and Mint2 shows that the N-terminal disordered sequences have a high net-positive charge. Plots were made with CIDER (Holehouse et al, 2015).

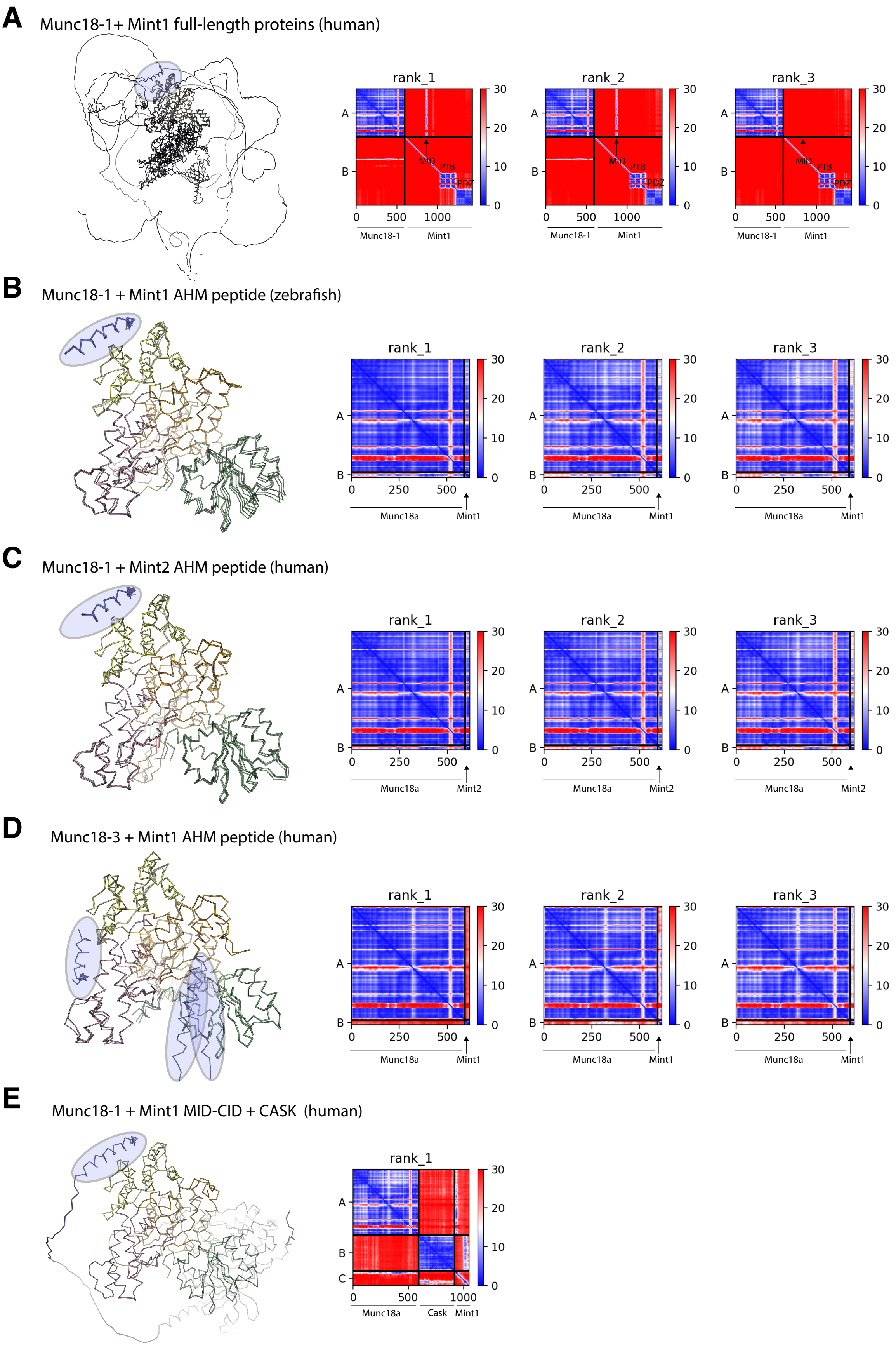

Figure S4. AlphaFold2 modelling of Munc18 interactions with Mint1. (Related to Fig. 3)
(A) Overlaid top three ranked models of human Mint1 bound to Munc18-1 from ColabFold shown in backbone ribbon representation. Mint1 is consistently modelled to bind Munc18-1 domain 3b via a short acidic α-helical motif (AHM) within its unstructured N-terminal domain (highlighted in blue). On the right hand side the predicted alignment error (PAE) is plotted for each model. Signals in the off-diagonal regions indicate strong structural correlations between residues in the peptide with the Munc18-1 protein. (B) Overlaid top three ranked models of the zebrafish Mint1 AHM sequence bound to Munc18-1 predicted with ColabFold. Similar to the human structures, the AHM is consistently modelled in an α-helical structure associated with the Munc18-1 domain3b (highlighted in blue). (C) Overlaid top three ranked models of the human Mint2 AHM sequence bound to Munc18-1 predicted with ColabFold. (D) Overlaid top three ranked models of the human Mint1 AHM sequence modelled with the non-binding Munc18-3 homologue. The AHM is modelled at several random positions (highlighted in blue). The PAE plots do not show evidence of significant interactions between the two proteins. (E) The top scoring model of the human Mint1 AHM and CID sequences bound to Munc18-1 and CASK using ColabFold. The AHM region is predicted in an identical binding site on Munc18-1 to the isolated peptide sequence (highlighted in blue). The CID sequence is predicted to bind to CASK in almost essentially the same conformation as the two previous crystal structures (Wu et al., 2020; Zhang et al., 2020).

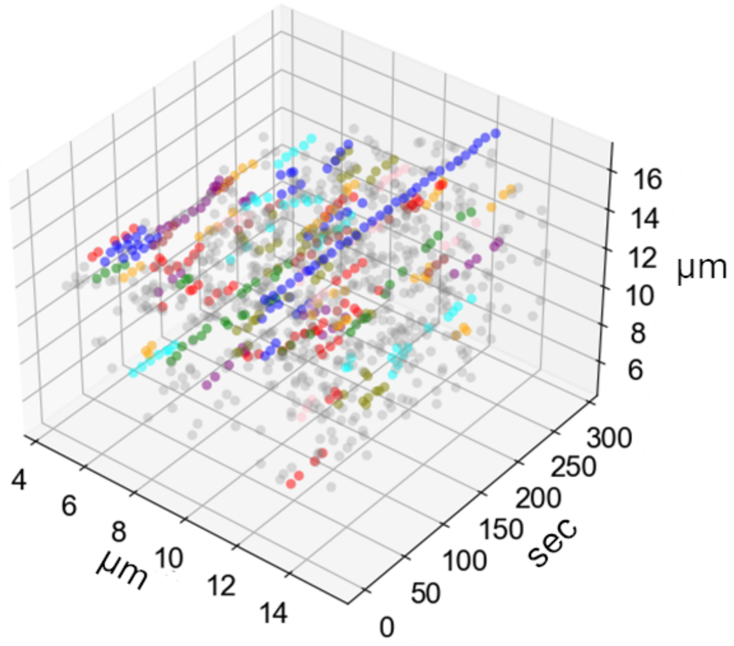

Figure S5. Analysis of cell footprint and fusion events in Munc18 DKO PC12 cells.
Representative fluorescent release event visualisation. The imaging dataset was divided into 10 sec (100 frame) intervals and the fluorescence averaged. Vesicle fusions at each interval were determined by Laplacian of Gaussian and used to create a 3D [x,y,t] array. DBSCAN was used to determine clusters of fluorescent areas/vesicles which persisted in the same area over time. A release event was defined as the end of each cluster.

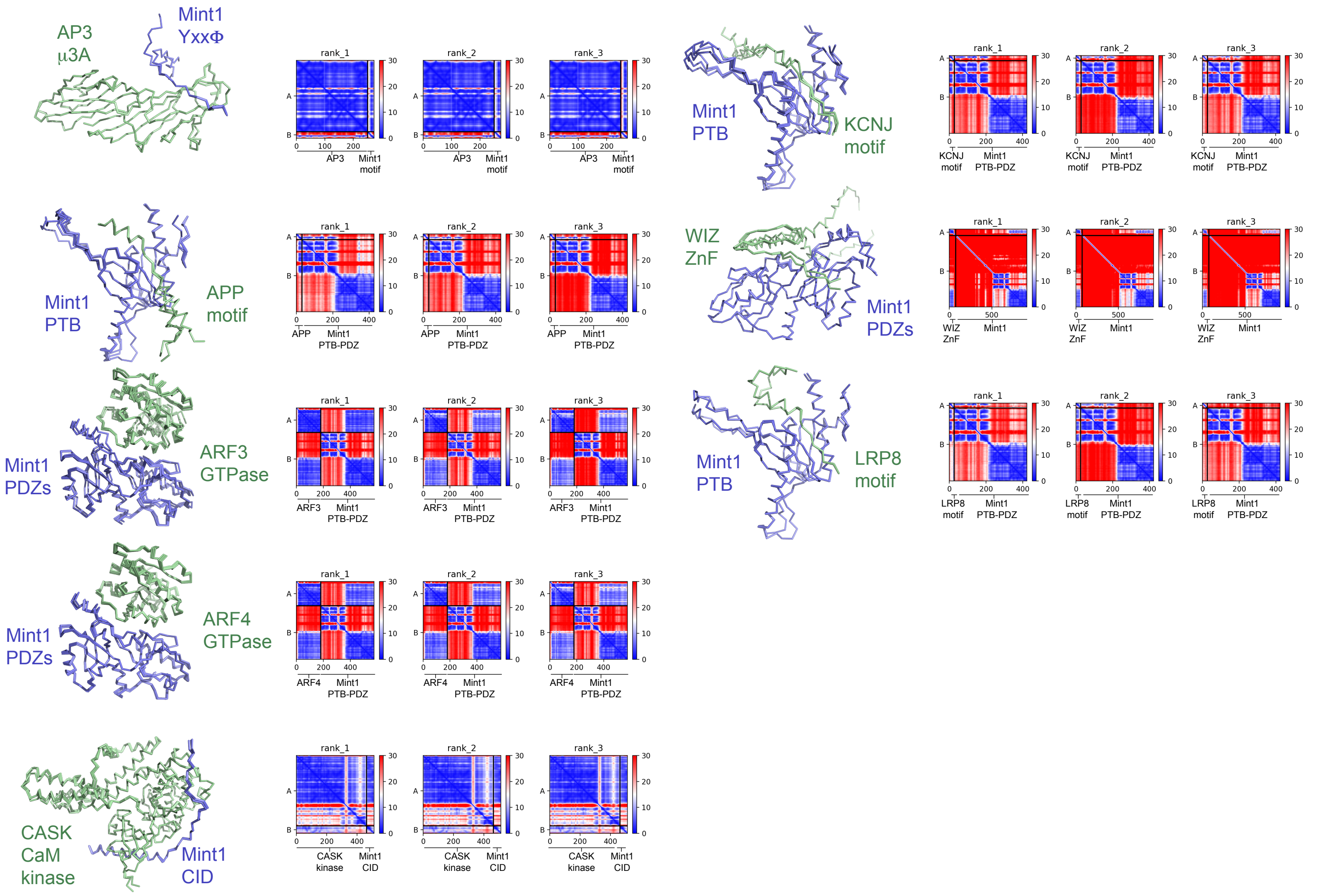

Figure S6. AlphaFold2 modelling of Mint1 interactions with proteins identified in BioGRID. (Related to Fig. 5 and 6)
Proteins identified in BioGRID as putative interactors of Mint1 and showing reasonable binding in AlphaFold2 predictions. The left panels show the top three ranked structures overlaid in backbone ribbon representation. On the right hand side, the predicted alignment error (PAE) is plotted for each model. Signals in the off-diagonal regions indicate strong structural correlations between residues in the peptide with the Mint1 protein. These focused predictions were performed on specific Mint1 domains with the ligand regions initially identified in high-throughput predictions of the two full-length proteins.

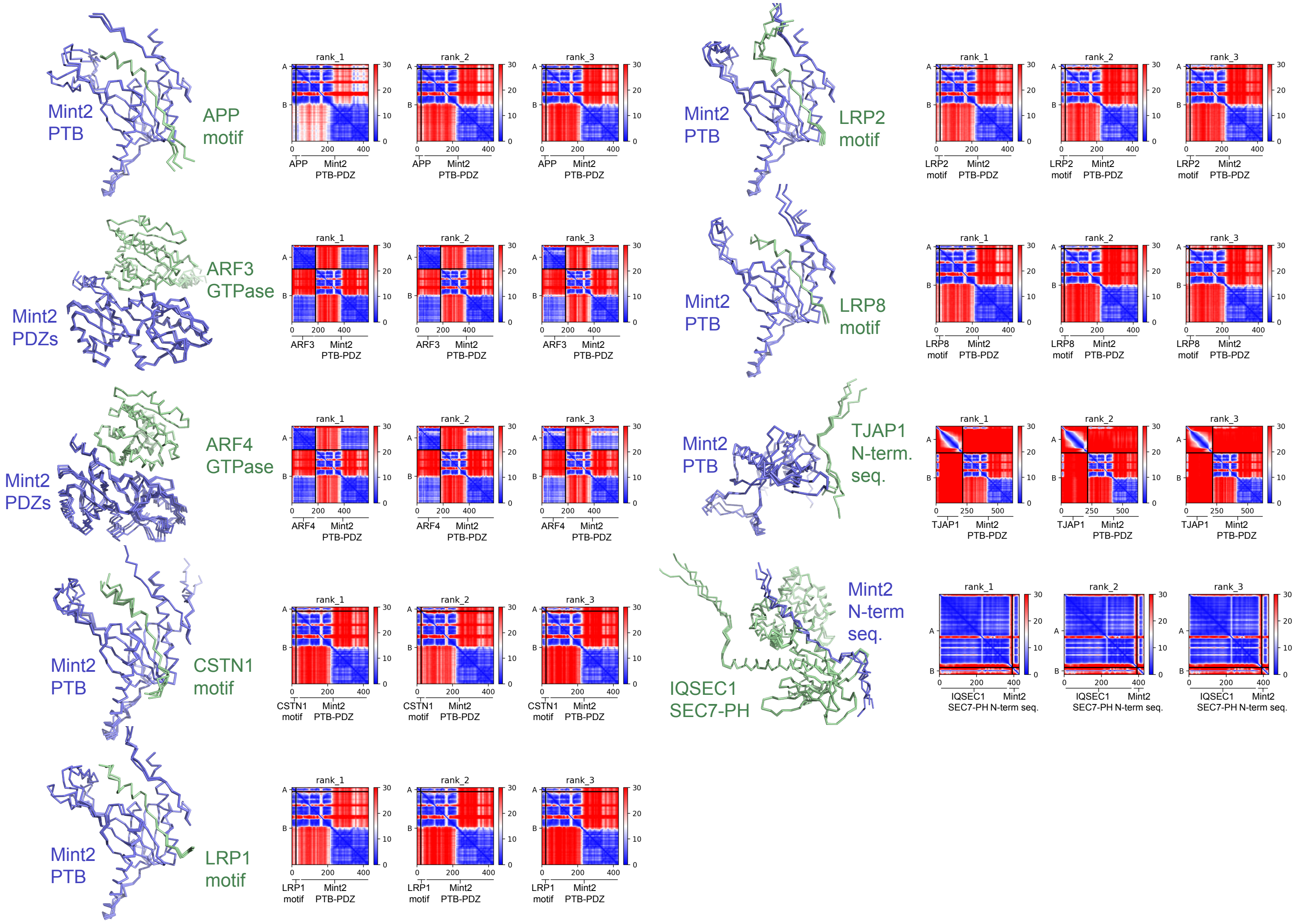

Figure S7. AlphaFold2 modelling of Mint2 interactions with proteins identified in BioGRID. (Related to Fig. 5 and 6)
Proteins identified in BioGRID as putative interactors of Mint2 and showing reasonable binding in AlphaFold2 predictions. The left panels show the top three ranked structures overlaid in backbone ribbon representation. On the right hand side the predicted alignment error (PAE) is plotted for each model. Signals in the off-diagonal regions indicate strong structural correlations between residues in the peptide with the Mint2 protein. These focused predictions were performed on specific Mint2 domains with the ligand regions initially identified in high-throughput predictions of the two full-length proteins.
